# Balance training improves memory and spatial cognition in healthy adults

**DOI:** 10.1038/s41598-017-06071-9

**Published:** 2017-07-18

**Authors:** Ann-Kathrin Rogge, Brigitte Röder, Astrid Zech, Volker Nagel, Karsten Hollander, Klaus-Michael Braumann, Kirsten Hötting

**Affiliations:** 1 Universität Hamburg, Department of Biological Psychology & Neuropsychology, Hamburg, Germany; 2 Friedrich Schiller University, Department of Human Movement Science, Jena, Germany; 3 Universität Hamburg, Department of Sports and Exercise Medicine, Hamburg, Germany

## Abstract

Physical exercise has been shown to improve cognitive functions. However, it is still unknown which type of exercise affects cognition. In the present study, we tested the hypothesis that a demanding balance training program improves memory and spatial cognition. Forty healthy participants aged 19–65 years were randomly assigned to either a balance or relaxation training intervention. Each group exercised twice a week for a total of 12 weeks. Pre- and posttests assessed balance performance, cardiorespiratory fitness, memory, spatial cognition, and executive functions. Only the balance group significantly increased in balance performance from pre- to posttest, while cardiorespiratory fitness remained unchanged in both groups. Moreover, the balance group significantly improved in memory and spatial cognition. Effects on executive functions were not observed. These results suggest that balance training is capable of improving particularly memory and spatial cognition. Therefore, an increase in cardiorespiratory fitness does not seem to be necessary to induce beneficial effects of physical exercise on cognition. It might be speculated that stimulating the vestibular system during balance training induces changes of the hippocampus and parietal cortex possibly via direct pathways between the vestibular system and these brain regions.

## Introduction

Developing methods to enhance neuroplasticity and cognitive functioning has become a major research interest of psychologists in the light of quickly advancing technologies and aging societies^1^. Among a large variety of behavioral interventions such as cognitive training programs and special nutrition, physical exercise programs have been suggested to improve cognition^2, 3^. Physical exercise over a course of several months has been shown to improve cognitive performance, including executive functions^4, 5^, speed of processing^6^, and memory^7^. Moreover, aerobic exercise has been found to slow down gray matter volume loss in the hippocampus and frontal lobes^8^. So far, most of the studies investigating the effects of physical exercise on cognitive functions have focused on aerobic training like running, walking and cycling. However, a recent meta-analysis on the effects of aerobic training on cognitive functions in older adults concluded that there is still no clear evidence for a causal link between an increase in cardiorespiratory fitness and cognitive benefits^9^. Thus, cardiorespiratory fitness improvements following aerobic training might only be one of multiple factors mediating the positive effects of exercise on cognition. This hypothesis is supported by recent reports suggesting beneficial effects of other types of exercise on cognitive functions. For example, randomized controlled intervention studies employing coordination training^5, 10^ and dancing^11^ reported positive effects on memory, selective attention, executive functions and spatial cognition compared to control groups.

Physical exercise, regardless of its aerobic or anaerobic metabolic demands, provides a stimulus to vestibular, neuromuscular and proprioceptive systems. The perception of self-motion and balance is coded by vestibular detection of inertial motion, in conjunction with proprioceptive and visual signals^12^. Connections between vestibular nuclei and the cerebellum, hippocampus, as well as prefrontal and parietal cortices provide information for cognitive functions such as spatial functions, navigation and memory^13, 14^. For example, bilateral vestibular lesions were found to result in decreased performance in spatial memory tasks^15^, hippocampal atrophy^16^, and reduced fractional anisotropy in white matter tracts within the limbic system and the thalamus^17^. Caloric stimulation of the vestibular nuclei in healthy adults, on the other hand, improved verbal and spatial memory^18^ and Galvanic vestibular stimulation modulated mental rotation and perspective taking abilities, depending on the threshold of the current^19, 20^.

It has been speculated that an increased stimulation of the vestibular system during self-motion might be an essential mediator between physical exercise and cognitive functioning^21^. Accordingly, animal studies have shown that improved balance performance resulted in higher survival rates of neurons^22^ and increased volume of the hippocampus and prefrontal areas^23^. In humans, balance skills have been associated with an increased volume of the hippocampus^24^, the basal ganglia^25^, and frontal and parietal brain areas^26^. However, data on the effects of balance training on cognitive functions, particularly related to memory and spatial cognition, are rare so far. A recent study in young adults found improvements in a spatial orientation task after one month of balance training^27^, compared to a passive control group.

The goal of the present study was to test the hypothesis that a physical exercise program with high demands on the vestibular system improves particularly memory and spatial cognition. To this end, we implemented a demanding balance training program in healthy adults in comparison to a relaxation training intervention. Both training types were expected to not affect cardiorespiratory fitness. Memory, spatial cognition and executive functions were assessed before and after the 12-week training program.

## Methods

### Participants

**Figure 1:**
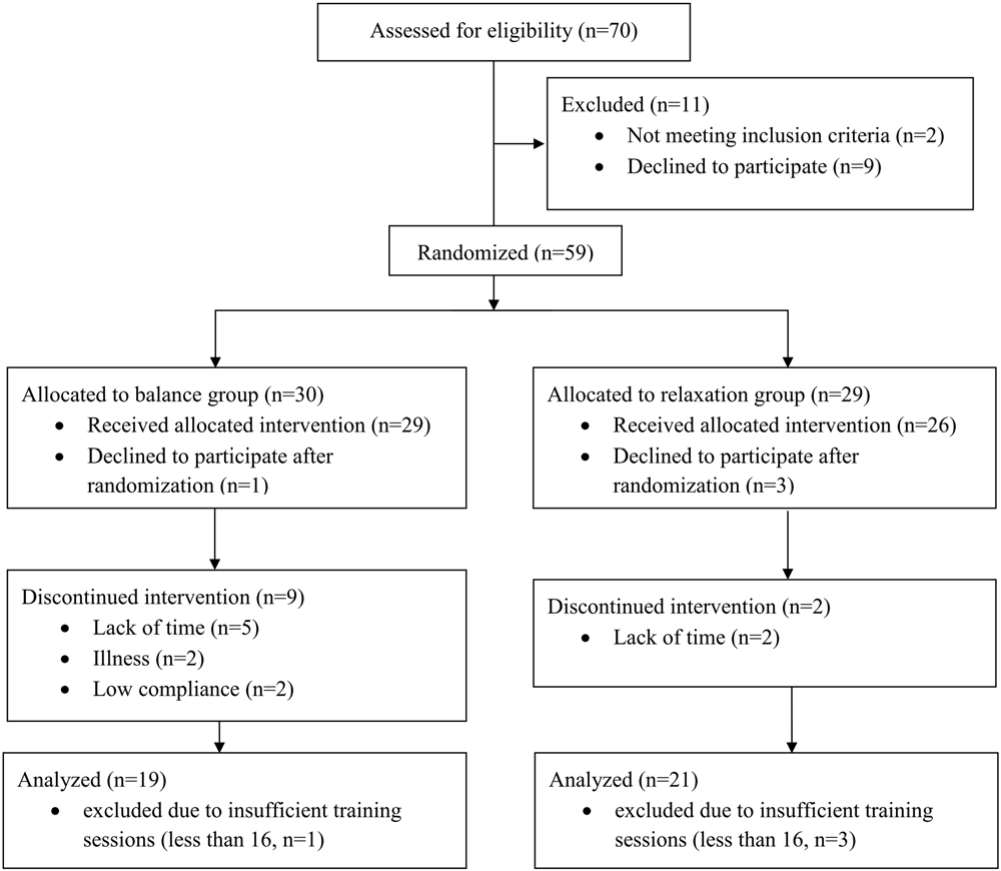
CONSORT diagram with participant flow.

A total of 70 healthy participants were recruited via public advertisements in the city of Hamburg (Germany). Participants between the age of 18 and 65 years were included if they reported sport activities of no more than five times a month during the last five years and no extensive experience in balance or relaxation training. All participants had normal or corrected-to-normal vision and normal hearing abilities. Exclusion criteria were untreated heart diseases, untreated respiratory diseases, neurological or psychiatric illnesses, acute musculoskeletal diseases or arthropathies. Additionally, participants underwent a sport medical examination. Based on meta-analysis of previous exercise-cognition studies^28^ we expected a medium effect size. Such an effect size can be statistically detected in a Time × Group design with a total sample size of 34 participants (power 0.80, alpha 0.05). A total of 40 participants successfully completed the study (for details, see Fig. 1). Dropout cases during the intervention are included in the participants’ characteristics at pretest to show that dropouts were not significantly different from participants completing the training (see Table 1).

**Table 1:** Participants characteristics at pretest (Mean, SD).

Measure	Balance group (n = 19)	Relaxation group (n = 21)	Balance vs. Relaxation p	Dropout (n = 23)	Training vs. Dropout* p
Age	43.9 (14.92)	46.6 (15.18)	0.59^c^	45.24 (15.02)	0.93^a^
Gender (female/male)	12/7	14/7	0.92^b^	16/9	0.93^b^
GSI	0.59 (0.44)	0.49 (0.31)	0.39^c^	0.54 (0.41)	0.65^a^
Vocabulary score	30.26 (4.51)	30.76 (3.85)	0.71^c^	29.19 (4.00)	0.56^a^
Body mass index	23.91 (2.21)	23.69 (3.80)	0.82^c^	24.62 (4.38)	0.71^a^
Self-reported physical activity (hours/week)	8.03 (7.18)	7.25 (3.73)	0.67^c^	6.20 (4.44)	0.56^a^
Self-reported physical activity (MET/week)	29.28 (23.49)	27.73 (14.77)	0.81^c^	22.54 (15.94)	0.82^a^
VO_2_peak (VO_2_ml/min/kg)	31.83 (5.27)	30.88 (8.95)	0.68^c^	29.03 (6.14)	0.60^a^
Training sessions total	20.63 (2.67)	21.24 (2.64)	0.48^c^		

*Note*. *Balance vs. Relaxation vs. Dropout comparison. ^a^ANOVA. ^b^Chi-square test. ^c^independent t-test. GSI = Global Severity Index of psychopathological symptoms. MET = Metabolic equivalents. VO_2_peak = Cardiorespiratory fitness.

Participants received a monetarily compensation of 130–156 € for participation, depending on the duration of the assessments. The study was approved by the ethical board of the German Psychological Society (DGPs) and carried out in accordance with the Helsinki Declaration guidelines. All participants gave written informed consent.

### Design

Participants were grouped into matched pairs based on age, gender and years of education. Participants of each pair were randomly assigned to the balance or the relaxation group. Each participant underwent three testing sessions prior and after the intervention. The test battery included balance tests, a cardiorespiratory fitness assessment, cognitive tests, as well as questionnaires on physical activity and psychopathological symptoms. All test assessors were blinded to the participant’s group assignment and participants were naïve to the study hypothesis.

### Intervention

Participants trained twice a week for 12 weeks in groups of 10–12 individuals, with each session lasting for 50 minutes. Both training groups were instructed by the same professional trainers. Each participant was supposed to take part in 24 training sessions. The training was offered for a total of 13 weeks, so that participants could catch up missed training sessions. All participants were advised not to change their habitual level of physical activity throughout the intervention period.

#### Balance training

Participants conducted a balance circuit training on varying surfaces, either on one leg or on both legs. They had to complete eight different balance stations per session, each lasting for 5 minutes. The training favored a situational approach: Tasks were designed to induce reactive postural adjustments, forcing participants to permanently re-stabilize. For example, one task was to keep balance in a single-leg stance while being consistently pulled to one side with a strong elastic strap around the hips. On half of the stations, working in tandems was required. For example, participants were standing on a wobble board, throwing a medicine ball to their partner and back while trying to regain balance. Exercises were progressively adjustable to the skill level of the participants by combining and increasing the difficulty of the balance components, i.e. by increasing the strap tension, the distance to the partner, or by closing the eyes while standing on one leg on a soft surface. No explicit strategies were taught. After six weeks, exercises were replaced with a new set, in order to keep the training interesting and sufficiently challenging.

#### Relaxation training

The relaxation group practiced two well-known relaxation techniques: Progressive muscle relaxation^29^ and autogenic training^30^. Participants were laying or sitting on mats. They were instructed to practice relaxation approaches by actively increasing and decreasing muscle tension of single body parts (progressive muscle relaxation) or by concentrating on the rhythm and depth of breathing (autogenic training). During the first half of the training period, progressive muscle relaxation was taught, and after six weeks, autogenic training was introduced to keep the participants’ motivation and attention high.

Both training groups were comparable with regard to duration, place, social contacts, group size and concentration on the body, yet only the experimental group received active balance training.

### Assessments

#### Physical assessments

Balance: To assess dynamic, functional and static balance parameters prior to and after the intervention, three different tests were used.

1. Dynamic balance was assessed with a stability platform (Stability Platform, Modell 16030 L, Lafayette Instrument Company, Lafayette, IN, USA). Participants stood barefoot on an unstable platform with a maximum deviation of 15° to each side of its horizontal alignment. They were asked to keep the platform in a horizontal position for 30 sec. A handrail was available to prevent falls, but during a trial participants placed their hands on their hips. After a one-minute practice trial, three trials per condition (eyes open and eyes closed) were conducted with rests of 30 sec in-between. Whether participants started with eyes open or with eyes closed was counter-balanced across participants. The test score was calculated as the mean time across trials the platform was in the horizontal position (±3° deviation).
2. To measure functional balance, the Balance Error Scoring System (BESS^31^ was used. Participants were tested barefoot with eyes closed using three different stances: double-leg stance (feet parallel to each other), single-leg stance (standing on the non-dominant leg) and a semi-tandem stance (non-dominant foot behind the dominant foot, heel-to-toe joint). During all trials, participants were instructed to stand as motionless as possible with their hands resting on their hips. The underground was either a firm surface or a 10 cm flat cushion of medium density foam (Airex® Balance-Pad, Gaugler & Lutz oHG, Germany). Following the BESS protocol, each position was tested twice on each underground; each trial lasted for 20 sec. Participants were video-recorded and two trained observers independently scored errors using a standardized rating scale. Error categories included opening eyes, lifting hands off the hips, stepping, stumbling or falling out of the position, lifting the forefoot or heel, abducting the hip by more than 30°, or failing to return to the starting position within 5 sec. To determine reliability, intraclass correlations (ICC) were calculated, ranging from r = 0.86 to r = 0.93 for each position and underground. For the overall score, the number of errors was summed up for each position and underground, but separately for the two repetitions. The mean of the two sums was used as the dependent variable.
3. A force plate (Type 9260AA6, Kistler® Instrumente GmbH, Switzerland) was used to assess postural sway velocity. For this, center of pressure data were collected during double leg, single leg and tandem leg stances, using the software BioWare (Kistler Instruments AG, version 4.0.1.2). Each position was tested three times with eyes closed and three times with eyes open. The order of eyes open/closed conditions was randomized across participants. Trials lasted 30 sec each; the last 20 sec of each trial were used for the analyses to avoid initial motion biases. A lowpass butterworth-filter implemented in MATLAB (The MathWorks Inc., USA) was used to preprocess the data. The sway velocity of the center of pressure (CoP) was determined by dividing the cumulative of medial–lateral and anterior–posterior CoP displacement by the trial time. The overall CoP score was calculated as the mean of the three trials per position, condition and sway axes (medial - lateral; anterior - posterior). Because the single-leg position was too difficult for most of the participants, only double leg and tandem-leg stances were included in the main score.

Cardiorespiratory fitness: Cardiorespiratory fitness was assessed by a graded maximal ergospirometry. Participants started on a cycle ergometer (ER 900, ergoline GmbH, Germany) with an initial workload of 50 Watt. The resistance was gradually increased, adding 50/3 Watt each minute. This procedure was continued until the subjectively perceived maximum exhaustion of the participant was reached. During the ergospirometry, oxygen uptake (COSMED, REF C09073-02-99), heart rate (CardioPart 12 Blue, Amedtec GmbH, Germany), capillary blood lactate (BIOSEN, EKF diagnostics GmbH, Germany), and blood pressure were monitored. Cardiorespiratory fitness was defined as the maximum oxygen uptake (ml/min) at exhaustion divided by body weight, hereinafter referred to as VO_2_peak (ml/min/kg).

#### Cognitive assessment

Memory: An auditory verbal paired-associate learning task was used to assess memory^32^. Twenty Polish-German word pairs (10 nouns and 10 verbs) were presented via speakers (Bose Companion 2 series II). The stimuli were recorded from female native speakers. The stimulus onset asynchrony (SOA) within word pairs was 2 sec, and 6 sec between word pairs. The stimuli were presented three times with a delay of 30 sec between blocks. The order of the vocabulary pairs were randomized within each block. After the learning phase, only the Polish words were presented in a random order and participants were asked to write down the German counterparts. The inter-stimulus-interval during the recall phase was set to 8 sec. The number of correctly recalled words was used as memory score. Two parallel versions of the test were administered. Participants were randomly assigned to one of two test versions at pretest and received the parallel version at posttest. One participant of the balance group was excluded from the memory test as he had polish language knowledge.

Spatial cognition tests:

1. Orienting and Perspective Taking Test (OPT^33^). The paper-pencil test assesses the ability to imagine scenes from different viewpoints. Participants were shown a picture with seven objects. The task was to imagine being at one object, facing another, and indicating the direction of a third object by drawing an angle estimate. There were 12 items on the same object ensemble to solve during a time limit of 5 min. The errors were determined by subtracting the participant’s angle estimate from the correct solution. The mean error score across items was calculated for each participant.
2. Figure orientation, subscale of the German Intelligence Structure Test (IST-2000R^34^). The test was presented in a paper-pencil version. Participants were given a set of 20 items, each consisting of different shapes that were cut into pieces. The aim was to mentally merge the pieces and to decide which of possible five shapes was present. The time limit was set to 7 min. Two parallel versions were used. Participants were randomly assigned to one of them at pretest and received the parallel version at posttest. The number of correctly solved items within the 7 min long testing phase was used as dependent variable.
3. Mirror images, subscale of the German “Wilde Intelligence Test” (WIT2^35^). Five identical but differentially rotated nonsense figures were presented; one was a mirror image of the same shape. Participant had to detect the latter. Participants had a time limit of 3 min for a total of 20 trials. Two parallel versions were used. Participants were randomly assigned to one of them at pretest and received the parallel version at posttest. The number of correctly solved items within the testing period of 3 min was used as dependent variable.

For further analyses, the results of the Orienting and Perspective Taking Test, the Figure Orientation Test and the Mirror Image Test were combined into one score: Each participants’ test score was standardized to the mean and standard deviation of the respective pretest score, separately for the pretest and posttest ((participant’s score at pretest or posttest - sample mean at pretest)/standard deviation at pretest). The mean of the normalized scores across the three tests defined the spatial cognition score of each participant.

Executive functions: A computer based German version of the Stroop Test^36^ was used to assess executive functions. The experiment was performed using the Presentation® software (Version 14.9, Neurobehavioral Systems, Inc., Berkeley, CA, USA). Color words were presented on a screen, either in a congruent or an incongruent font color (red, yellow, blue green). With a short delay (SOA = 300 ms), a second color word was shown underneath, written in gray font color. Participants had to decide whether or not the font color of the upper word was congruent with the meaning of the color word written in gray font by pressing one of two buttons as fast as possible (yes = left, no = right). The two color words were presented for 1000 ms, followed by a black screen. The new fixation cross followed by the next stimulus pair was presented 1000 ms after the participant’s button press. Additionally, non-color adjectives (i.e. “empty”, “high”) printed in color were displayed as a neutral control condition. Forty-eight trials per condition (incongruent, congruent, and neutral) were presented in a randomized order. Incorrect trials, and trials with reaction times <200 ms and >3 SD above the group mean per condition were eliminated. The inference score was defined as the mean reaction time for the incongruent condition minus the mean reaction time for the congruent condition.

Participants were familiarized with the task within a 20 trials practice run in which immediate feedback was presented after every trial. During the main test, no feedback was given. Three participants did not complete the Stroop Test due to being color-blind (n = 2); one was a non-native German speaker.

#### Further assessments

We assessed participants’ vocabulary, physical activity and general psychopathological symptoms with questionnaires. These assessments allowed us to control for potential impact of these variables on the above described cognitive functions as well as their change across the intervention phase.

The German “Mehrfachwahl-Wortschatztest” (MWT-B^37^) was used to estimate verbal intelligence. The participant had to find the item out of five which was an existing German word. The four distractors were pronounceable German pseudo-words of approximately the same length. The test is sensitive to the educational background and correlates with the global IQ in healthy adults.

The “Freiburger Questionnaire on Physical Activity” (FQPA^38^) was used to assess the overall self-reported physical activity at pretest and after three months. The questionnaire covers everyday physical activities such as taking the stairs, walking to work etc., as well as leisure time and sport activities. Hours of total activity per week were additionally converted into metabolic equivalents (MET) to estimate total energy expenditure associated with physical activities^39^.

Self-reported psychopathological symptoms were assessed with the Symptom Checklist-90-R (SCL 90R^40^). The psychometric questionnaire with 90 items on 9 subscales aims to evaluate a broad range of psychological strains and psychopathology. The global score (Global Severity Index, GSI) based on all subscales was used to compare participant’s psychopathological symptoms across groups and possible changes after the intervention.

### Data analysis

The data analysis was performed using R, version 3.3.1^41^. Intervention effects were compared by means of ANCOVA: In the linear models, posttest scores were compared between groups, adjusted for pretest scores and age which were included as covariates^42^. The results will be displayed as the pretest- and age-adjusted group difference at posttest, along with the corresponding 95% confidence intervals (CI) and Cohens *d* as standardized effect size. The overall α level was set to p < 0.05.

Technical problems and interruption during the assessment led to missing data for four single tests (memory: n = 1, OPT: n = 1, IST: n = 1, VO_2_peak: n = 2). Multiple Data Imputation (R package MICE 2.0^43^) was used to replace these missing data. For the analyses, 10 imputation models were estimated separately (specification = predictive mean matching). The different estimates were pooled into one model, which is reported in the results section. Associations between changes from pre-to posttest and age were computed using Pearson correlations.

#### Data availability

The datasets generated during the present study are available from the corresponding author on reasonable request.

## Results

Participants of the balance and the relaxation group did not differ with respect to age, gender, vocabulary score, cardiorespiratory fitness, self-reported physical activity, and psychopathological symptoms (see Table 1). Moreover, the groups did not differ significantly with respect to the physical and cognitive variables at pretest (all p > 0.250, see Table 2).

**Table 2:** Physical and cognitive variables: Means (SD) at pre- and posttest separately for the balance and the relaxation group.

Test	Time	Balance group	Relaxation group	Adjusted group difference at posttest [95% CI], Cohens *d*
Stability platform	Pretest	5.40 (1.17)	5.73 (1.85)	**1**.**57 [0**.**49**, **2**.**65]**, *d* = 0.66
	Posttest	7.54 (2.57)	6.29 (1.84)	
BESS	Pretest	15.24 (9.14)	17.78 (8.25)	−0.52 [−0.3.25, 2.22], *d* = −0.09
	Posttest	13.63 (8.68)	16.12 (7.38)	
CoP	Pretest	0.55 (0.07)	0.59 (0.12)	−0.001 [−0.08, 0.08], *d* = −0.01
	Posttest	0.63 (0.11)	0.67 (0.18)	
VO_2_peak	Pretest	31.83 (5.27)	30.883 (8.95)	−0.83 [−3.49, 1.83], *d* = −0.14
	Posttest	32.07 (6.52)	31.947 (7.89)	
Memory	Pretest	7.50 (5.34)	7.67 (4.03)	**1**.**60 [0**.**05**, **3**.**05]**, *d* = 0.47
	Posttest	9.44 (5.48)	8.00 (4.16)	
Spatial Score	Pretest	0.001 (0.80)	−0.013 (0.77)	**0.31 [0**.**04**, **0.58]**, *d* = 0.51
	Posttest	0.443 (0.75)	0.040 (0.91)	
Stroop	Pretest	229.88 (132.27)	255.59 (147.88)	−3.47 [−66.39, 59.44], *d* = −0.03
	Posttest	190.55 (81.87)	206.03 (120.41)	
Self-reported physical activity (hours/week)	Pretest	8.03 (7.18)	7.25 (3.73)	−1.12 [−4.62, 2.39], *d* = −0.17
	Posttest	9.77 (6.61)	10.44 (6.89)	
Self-reported physical activity (MET/week)	Pretest	29.28 (23.49)	27.72 (14.77)	1.37 [−12.03, 14.78], *d* = 0.05
	Posttest	35.86 (24.27)	33.44 (24.43)	
GSI	Pretest	0.59 (0.44)	0.49 (0.31)	2.68 [−2.95, 8.31], *d* = 0.16
	Posttest	0.45 (0.33)	0.35 (0.22)	

*Note*. Bold print differences denote significant results. GSI = Global Severity Index of psychopathological symptoms. MET = Metabolic equivalents, CoP = Center of pressure velocity.

### Physical Variables

**Figure 2:**
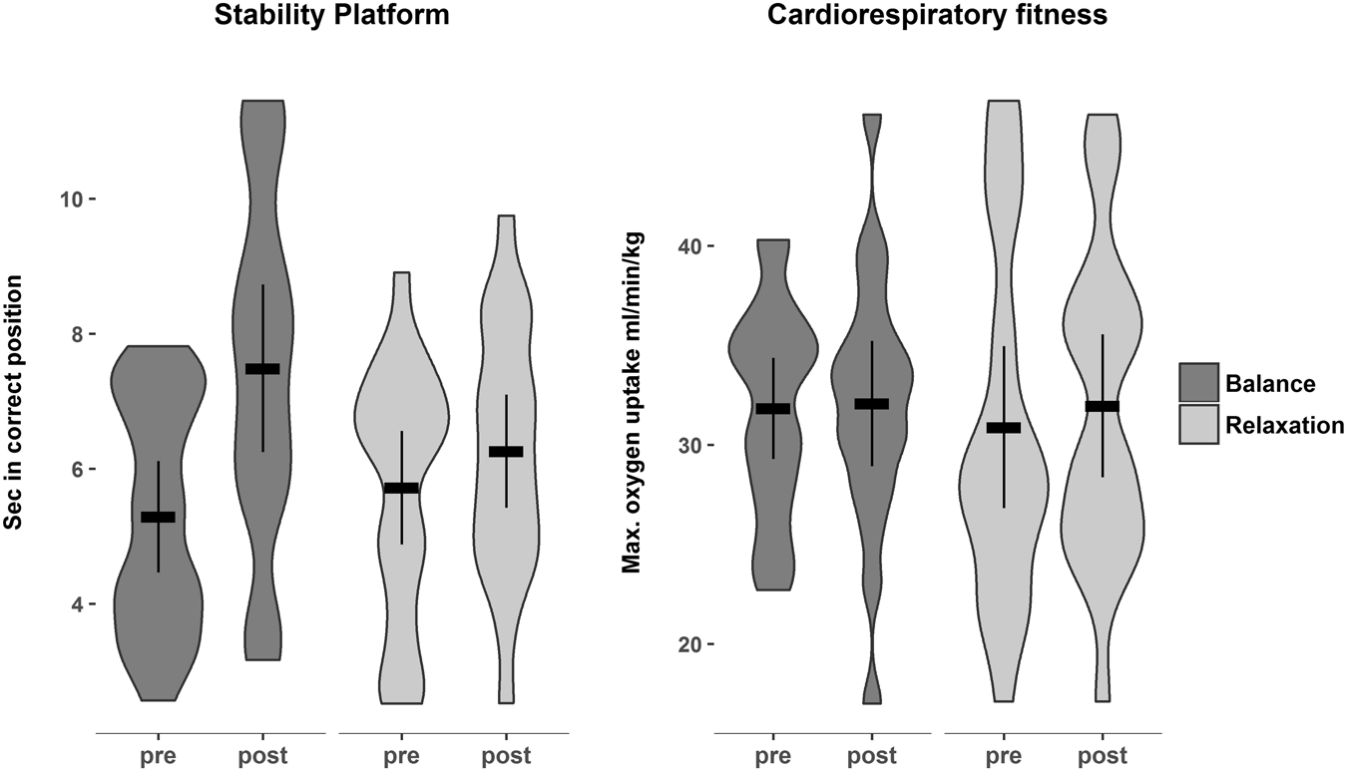
Violin plots for the dynamic balance performance (stability platform) and cardiorespiratory fitness for the balance (dark gray) and the relaxation group (light gray) at pre- and posttest, showing the distribution and density of the data. Horizontal bars indicate the group mean. Error bars indicate 95% confidence intervals.

The balance training increased participants’ dynamic balance performance on the stability platform as indicated by a significant effect of group, *F*(1, 36) = 8.72, *p* = 0.005. The mean of the balance training group at posttest was higher than the mean of the relaxation group (see Fig. 2, group difference = 1.57, 95% CI = [0.49, 2.65], *d* = 0.66).

There was no significant training effect on neither the CoP sway velocity as assessed with the forceplate, *F*(1, 36) < 0.001, *p* = 0.962, group difference = −0.001, 95% CI = [−0.08, 0.08], *d* = −0.01, nor on functional balance, as measured with the BESS, *F*(1, 36) = 0.16, p = 0.704, group difference = −0.52, 95% CI = [−0.3.25, 2.22], *d* = −0.09.

The training did not increase participants’ cardiorespiratory fitness. There was no significant effect of group for VO_2_peak: *F*(1, 36) = 0.40, *p* = 0.531, group difference = −0.83, 95% CI = [−3.49, 1.83], *d* = −0.14, see Fig. 2.

### Cognitive Variables

**Figure 3:**
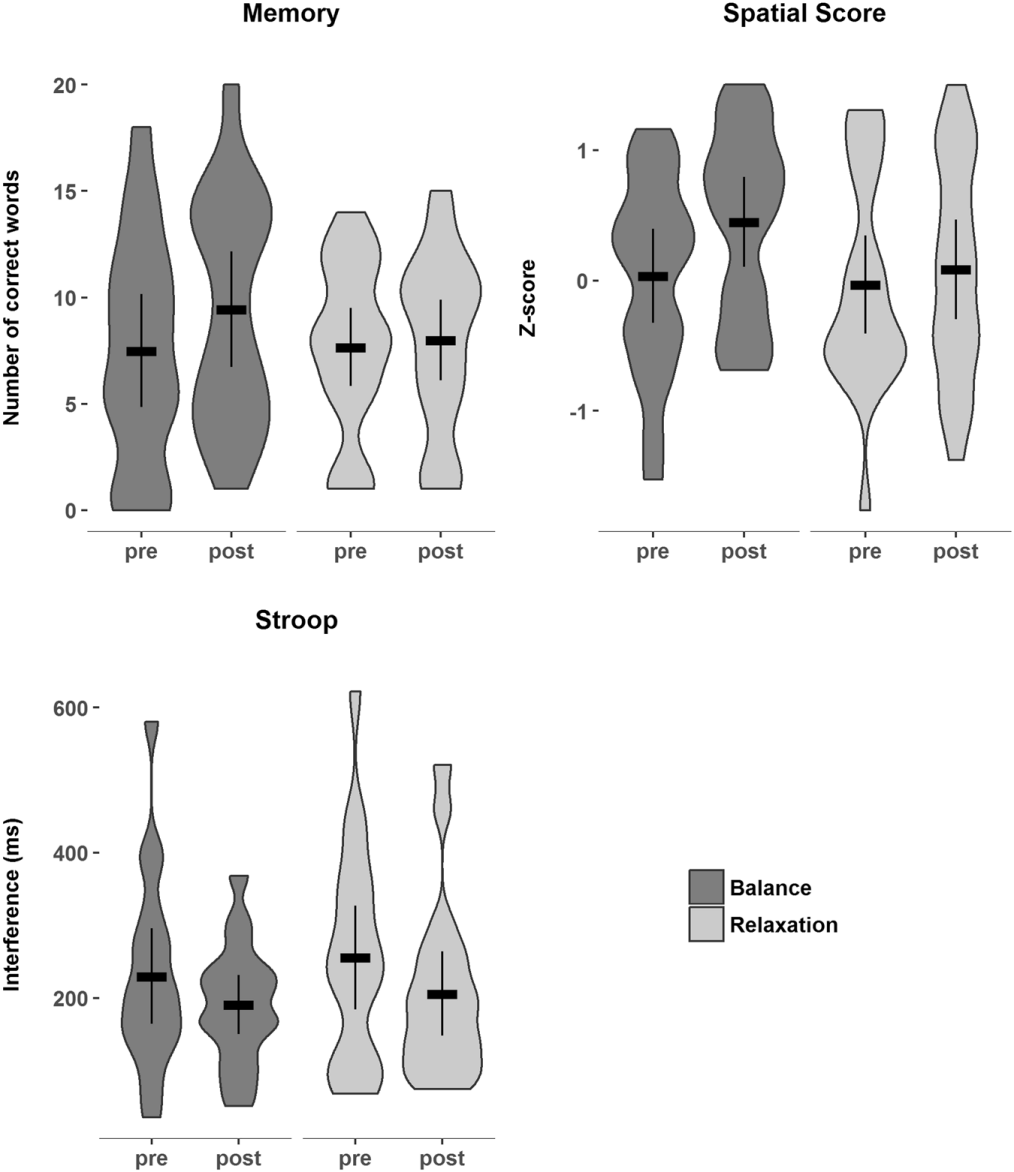
Violin plots of the performance of the balance group (dark gray) and the relaxation group (light gray) for the memory test, spatial score and executive functions (Stroop test), showing the distribution and density of the data. Horizontal bars indicate the group mean. Error bars indicate 95% confidence intervals.

A significant effect of group, *F*(1, 35) = 4.40, *p* = 0.043, was found for the memory score. After training, the adjusted mean of the balance group was higher than the mean of the relaxation group (group difference = 1.60, 95% CI = [0.05, 3.05], *d* = 0.47, see Fig. 3).

The analysis of spatial score yielded a significant effect of group, *F*(1, 36) = 5.28, *p* = 0.027. After training, the adjusted spatial score was higher in the balance than in the relaxation training group (group difference = 0.31, 95% CI = [0.04, 0.58], *d* = 0.51, see Fig. 3).

To test whether the Stroop effect could reliably be elicited to measure executive functions in the present study, reaction times of congruent trials were compared to reaction times of incongruent trials, collapsed across groups and time. As expected, participants reacted faster in the congruent condition (M = 664.774 ms, SD = 177.583 ms) compared to the incongruent condition (M = 885.564, SD = 239.654 ms), *t*(134) = 6.37, *p* < 0.001. To analyze training effects on executive functions, an interference score was calculated by subtracting the mean reaction time of the incongruent condition from the mean reaction time of the congruent condition for each participant. There was no significant effect of group on the Stroop interference score at posttest, *F*(1, 33) = 0.01, *p* = 0.911, group difference = −3.47, 95% CI = [−66.39, 59.44], *d* = −0.03 (see Fig. 3).

### Self-reported physical activity and psychopathological symptoms

On average, participants increased their total weekly physical activity from M = 7.62 (SD = 5.58) hours at pretest to M = 10.12 (SD = 6.68) hours at posttest. Moreover, they expended more energy per week (MET score) at posttest (M = 34.59, SD = 24.07) than at pretest (M = 28.45, SD = 19.16). The average increase in self-reported physical activity reflects the participation in the supervised training, i.e. two hours/week with low to moderate intensity. Importantly, for the self-reported physical activity, the analysis yielded no significant effect of groups after training: Neither for hours/week: *F*(1, 36) = 0.42, *p* = 0.521, group difference = −1.12, 95% CI = [−4.62, 2.39], *d* = −0.17 nor for MET: *F*(1, 36) = 0.04, *p* = 0.84, group difference = 1.37, 95% CI = [−12.03, 14.78], *d* = 0.05, respectively (see Table 2).

The analysis of the psychopathological symptoms assessed with the SCL-90 displayed no significant effect of group, *F*(1, 37) = 0.42, *p* < 0.521, group difference = 0.035, 95% CI = [−0.07, 0.14], *d* = 0.15, see Table 2).

Age did not correlate with changes from pre- to posttest in any of the physical and cognitive variables (all r < 0.12, p > 0.250), indicating that younger and older participants did not differ in their benefits after training.

## Discussion

The goal of the present randomized controlled intervention study in a group of healthy adults was to test the hypothesis that balance training improves cognitive functions, in particular memory and spatial cognition. Dynamic balance performance improved only in the balance group from pre- to posttest. By contrast and as expected, changes in cardiorespiratory fitness (VO_2_peak) were observed neither in the balance group nor in the relaxation group. Only the balance group improved in memory and spatial cognitive abilities. Finally, none of the two groups displayed changes in executive functions.

The findings suggest that systematic balance training is capable of enhancing some cognitive functions, such as memory and spatial cognition. Crucially, an increase in cardiorespiratory fitness does not seem to be necessary for eliciting beneficial effects of physical exercise on cognitive functions. This pattern implies multiple mechanisms for physical activity affecting cognitive functions.

In the present study, we used a balance training in order to particularly engage the vestibular system. The vestibular system essentially contributes to spatial cognition and orientation by detecting linear acceleration during self-motion^21^. Moreover, the vestibular system has anatomical connections to the medial-temporal lobe as well as to parieto-temporal cortical networks which are known to be involved in spatial navigation^13^. Structural changes after short-term balance training were found in frontal and parietal areas^26^. Moreover, professional dancers and slackliners were found to have larger gray matter volumes in the posterior hippocampus but smaller volumes in the anterior hippocampus compared to non-balance experts^24^. The authors interpreted the structural differences as indicator of plastic changes following perseverative stimulation of the vestibular-visual pathways to the hippocampus. The behavioral improvements in memory and spatial cognition after 12 weeks of balance training of the present study are in accord with these structural data. The balance training group improved in a paired-associative learning task which is associated with the hippocampal memory system^44^. Perspective taking abilities and mental rotation tasks seem both to rely on bilateral parietal cortices and the hippocampus^45^, respectively.

We did not find improvements of executive functions after the balance (and relaxation) training. Results of a study comparing coordination, aerobic and stretching training in older adults reported benefits in both physically active training groups on executive functions^5^. The coordination training in the study of Voelcker-Rehage *et al*. focused on fine and gross motor coordination rather than on balance like the present study. The effects of balance training might be more restricted to memory and spatial functioning. Improvements in executive functions in studies implementing aerobic exercise programs have been repeatedly observed^28^. In the present study, participants underwent a graded ergospirometry before and after the intervention to control for possible changes in cardiorespiratory fitness. Neither the balance group nor the relaxation group significantly changed in cardiorespiratory fitness.

In addition, participants of the present sample were on average younger and the age range was much broader than in previous studies^46^. It has been hypothesized that cognitive functions are particularly susceptible to treatment such as physical exercise interventions, when they undergo age-related change^2^. Therefore, another reason for lacking a significant effect in executive functions might be the broad age range of the present sample. We did not find differences in the performance change between younger and older participants, neither in physical nor in cognitive variables. Future studies should comprise larger sample sizes to investigate possible differences between age groups with a higher power.

The present findings demonstrate beneficial effects of balance training, compared to relaxation training, on memory and spatial cognition. In order to conclude that group differences at posttest are explained by the specific balance training, possible alternative accounts need to be discussed: Balance training comprises proprioceptive, visual and motor learning to a larger degree than relaxation training. The vestibular neural pathways are inherently multisensory, and vestibular signals converge with proprioceptive, visual and tactile information at early stages, such as brainstem vestibular nuclei and deep cerebellar fastigial nuclei in the afferent pathway^12^. Whether one of the sub-processes or rather the integration of vestibular, somatosensory and visual signals are essential for the training induced increase in memory and spatial cognition, must be addressed in future studies. Tasks requiring coordination with a partner and objects were introduced to make the training diversified and more appealing for the participants. Although the relaxation group acquired two different relaxation techniques, overall task complexity was higher in the balance training group. We cannot fully exclude the possibility that complexity as such improved performance in the experimental group, though we consider this account as unlikely: Practicing to handle and coordinate more complex task demands is a typical executive function, rather than being specifically linked to memory and spatial skills. However, groups did not differ in executive functions at posttest.

Physical activity is known to have antidepressant and anxiolytic effects and to increase resilience to stress^47^. In the present study, a global score of psychopathological symptoms was assessed at baseline and after the intervention. Importantly, the score did not differ between groups at pretest, nor did groups differ in their change from pretest to posttest, rendering it unlikely that a reduction in psychological distress might account for the present findings. Additionally, both training groups displayed the same level of self-reported habitual physical activity (MET) over time. The increase of two additional hours of physical activity per week at posttest compared to pretest was recorded in both groups and reflected the participation in the intervention. Therefore, the observed improvements in memory and spatial cognition are likely related to the balance intervention.

In sum, we are able to conclude that 12 weeks of balance training in healthy adults has positive effects on memory and spatial cognition, and an increase in cardiorespiratory fitness does not seem to be necessary to induce beneficial effects of physical exercise on cognition.

From an applied perspective, balance training might represent a promising alternative intervention for individuals who are not able to participate in aerobic training following health restrictions.
