# Author Correction: Balance training improves memory and spatial cognition in healthy adults

**DOI:** 10.1038/s41598-018-35957-5

**Published:** 2018-11-22

**Authors:** Ann-Kathrin Rogge, Brigitte Röder, Astrid Zech, Volker Nagel, Karsten Hollander, Klaus-Michael Braumann, Kirsten Hötting

**Affiliations:** 1 Universität Hamburg, Department of Biological Psychology & Neuropsychology, Hamburg, Germany; 2 Friedrich Schiller University, Department of Human Movement Science, Jena, Germany; 3 Universität Hamburg, Department of Sports and Exercise Medicine, Hamburg, Germany

Correction to: *Scientific Reports* 10.1038/s41598-017-06071-9, published online 18 July 2017

This Article contains errors in the Methods section under subheading ‘Assessments’.

“A force plate (Type 9260AA6, Kistler® Instrumente GmbH, Switzerland) was used to assess postural sway velocity.”

should read:

“A force plate (Type 9260AA6, Kistler® Instrumente GmbH, Switzerland) was used to assess postural sway.”

“The sway velocity of the center of pressure (CoP) was determined by dividing the cumulative of medial–lateral and anterior–posterior CoP displacement by the trial time.”

should read:

“Center of pressure (CoP) was determined by the cumulative of medial–lateral and anterior–posterior CoP displacement.”

This Article also contains an error in the Results section under subheading ‘Physical Variables’.

“There was no significant training effect on neither the CoP sway velocity as assessed with the forceplate, F(1,36) < 0.001, p = 0.962, group difference = −0.001, 95% CI = [−0.08, 0.08], d = −0.01, nor on functional balance, as measured with the BESS, F(1, 36) = 0.16, p = 0.704, group difference = −0.52, 95% CI = [−0.3.25, 2.22], d = −0.09.”

should read:

“There was no significant training effect on neither the CoP as assessed with the forceplate, F(1,36) < 0.001, p = 0.962, group difference = −0.001, 95% CI = [−0.08, 0.08], d = −0.01, nor on functional balance, as measured with the BESS, F(1, 36) = 0.16, p = 0.704, group difference = −0.52, 95% CI = [−0.3.25, 2.22], d = −0.09.”

This Article also contains an error in the title of Table 2.

“Physical and cognitive variables: Means (SD) at pre- and posttest separately for the balance and the relaxation group. *Note*. Bold print differences denote significant results. GSI = Global Severity Index of psychopathological symptoms. MET = Metabolic equivalents, CoP = Center of pressure velocity.”

should read:

“Physical and cognitive variables: Means (SD) at pre- and posttest separately for the balance and the relaxation group. *Note*. Bold print differences denote significant results. GSI = Global Severity Index of psychopathological symptoms. MET = Metabolic equivalents, CoP = Center of pressure.”

